# Implementation of guidelines on prevention of coercion and violence: baseline data of the randomized controlled PreVCo study

**DOI:** 10.3389/fpsyt.2023.1130727

**Published:** 2023-05-11

**Authors:** Sophie Hirsch, Johanna Baumgardt, Andreas Bechdolf, Felix Bühling-Schindowski, Celline Cole, Erich Flammer, Lieselotte Mahler, Rainer Muche, Dorothea Sauter, Angelika Vandamme, PreVCo Study Group, Tilman Steinert

**Affiliations:** ^1^Department for Psychiatry and Psychotherapy I, Faculty of Medicine, Ulm University, Ulm, Germany; ^2^Department for Psychiatry and Psychotherapy Biberach, ZfP Südwürttemberg, Biberach, Germany; ^3^Department of Psychiatry, Psychotherapy and Psychosomatics, Vivantes Hospital Am Urban, Berlin, Germany; ^4^Research Institute of the Local Health Care Funds (WIdO), Berlin, Germany; ^5^Department of Psychiatry and Psychotherapy, Charité Univesitätsmedizin, Berlin, Germany; ^6^Department of Psychiatry and Psychotherapy, Clinics in the Theodor-Wenzel-Werk, Berlin, Germany; ^7^Faculty of Medicine, Institute of Epidemiology and Medical Biometry, Ulm University, Ulm, Germany

**Keywords:** psychiatry, seclusion, restraint, guidelines, evidence based care, implementation, mental heath, coercion

## Abstract

**Introduction:**

The PreVCo study examines whether a structured, operationalized implementation of guidelines to prevent coercion actually leads to fewer coercive measures on psychiatric wards. It is known from the literature that rates of coercive measures differ greatly between hospitals within a country. Studies on that topic also showed large Hawthorne effects. Therefore, it is important to collect valid baseline data for the comparison of similar wards and controlling for observer effects.

**Methods:**

Fifty five psychiatric wards in Germany treating voluntary and involuntary patients were randomly allocated to an intervention or a waiting list condition in matched pairs. As part of the randomized controlled trial, they completed a baseline survey. We collected data on admissions, occupied beds, involuntarily admitted cases, main diagnoses, the number and duration of coercive measures, assaults and staffing levels. We applied the PreVCo Rating Tool for each ward. The PreVCo Rating Tool is a fidelity rating, measuring the degree of implementation of 12 guideline-linked recommendations on Likert scales with a range of 0–135 points covering the main elements of the guidelines. Aggregated data on the ward level is provided, with no patient data provided. We performed a Wilcoxon signed-rank-test to compare intervention group and waiting list control group at baseline and to assess the success of randomization.

**Results:**

The participating wards had an average of 19.9% involuntarily admitted cases and a median 19 coercive measures per month (1 coercive measure per occupied bed, 0.5 per admission). The intervention group and waiting list group were not significantly different in these measurements. There were 6.0 assaults per month on average (0.3 assaults per occupied bed and 0.1 per admission). The PreVCo Rating Tool for guideline fidelity varied between 28 and 106 points. The percentage of involuntarily admitted cases showed a correlation with coercive measures per month and bed (Spearman’s Rho = 0.56, *p* < 0.01).

**Discussion:**

Our findings that coercion varies widely within a country and mainly is associated with involuntarily admitted and aggressive patients are in line with the international literature. We believe that we included a sample that covers the scope of mental health care practice in Germany well.

**Clinical trial registration**: www.isrctn.com, identifier ISRCTN71467851.

## Introduction

The study on the implementation of the German guidelines on Prevention of Coercion (PreVCo study, German acronym IVZ-S3) intends to investigate the extent to which a structured, operationalized implementation of the guidelines and an external consultation improve guideline-compliant working and an improvement of patient-related outcomes (less coercion without an increase in assaults). The German guidelines on the prevention of coercion and the prevention and treatment of aggressive behavior in psychiatry contain a total of 83 evidence- or consensus-based recommendations and 6 statements (1). These have been condensed into 12 recommendations for action (2). To this end, PreVCo, a randomized, 1:1 matched study is conducted on 55 across Germany psychiatric wards treating both voluntarily and involuntarily admitted patients. Background, rationale and study design of the PreVCo study have been published before the start of the study (3).

Nowadays, there is a general consensus that coercive measures such as restraint or seclusion of a service user should only be used as a last resort if, despite attempts at de-escalation and offers of treatment, there is still an acute danger to the patient or others (4). Therefore, the prevention of coercion has been the goal of many initiatives and programs, whose efficacy was demonstrated in several studies (5–8). However, whether the following recommendations derived from such studies also lead to the prevention of coercive measures in everyday clinical practice is largely unclear. To date, there have been few studies of guideline implementation in psychiatry (9, 10) and even fewer which actually examine patient-related outcomes (11). Follow-up studies examining psychiatric wards that have participated in coercion prevention trials years or decades later, however, give reason for hope: the implementation of the Engagement Model could reduce coercion sustainably up to 13 years (12). Implementing interventions focusing on the physical environment could stabilize low numbers of coercive measures over 10 years (13, 14).

In a study in which wards started interventions to reduce coercion at different points in time, it was found that already by participating in the study, even without starting an intervention, a considerable reduction in coercion could be achieved (15). This seems to be most likely explained by observer effects also known as the *Hawthorne effect* (16, 17). Therefore, it seemed particularly important to introduce a survey of baseline conditions in the PreVCo study, in which, as under study conditions, monitoring and documentation is already carried out.

Another problem is that due to the clinical and scientific focus on the prevention of coercive measures, as well as other forms of formal and informal coercion, the handling of aggressive behavior in psychiatry is increasingly discussed. In particular, healthcare workers and their representatives (e.g., unions) question the extent to which the prevention of coercion is bought with increased assaults on staff and patients (18). Therefore, assaults must also be recorded at baseline.

Capturing and publishing baseline characteristics are becoming more common (19, 20) and are considered to be good research standard in the progress of publishing multicenter RCTs. Baseline expression is an important predictor of expression post-intervention, as there is often a strong internal correlation. On the other hand, by controlling for baseline expression, interesting secondary analyses can be performed and baseline characteristics can be found that predict a particularly good effectiveness of the intervention (e.g., 21, 22). Since the mental health system has been intensively dealing with the topic of coercion for several years, and there were predecessor guidelines on the topic, it could be assumed that many wards had already implemented some of the 12 recommendations, at least in part. Hence, as the aim of the PreVCo study is to investigate the efficacy of guideline implementation on the use of coercion, an objective of this baseline assessment was to analyze whether the level of pre-existing guideline adherence at baseline predicts the use of coercion in the participating wards.

Baseline data can help to describe the population that is not yet affected by the intervention under investigation to verify, for example, the severity of symptoms in the study population and the representativeness of a clinical population (e.g., for mental health (23–25)). Moreover, this approach can provide arguments for good external validity of a study (e.g., for mental health (26, 27)). Baseline data can also be interesting when little is known about the population so far or when the population is very heterogeneous in terms of outcome, which is the case for restraints, as shown by epidemiological studies (28).

Baseline data provide information on whether randomization worked and whether groups were indeed comparable before intervention, which is not the case in many studies, as a recent review showed (29). By rigorously collecting and publishing baseline data in the scientific community, different randomization and matching procedures can be compared and an appropriate procedure can be selected for the study (30). Experts can get a picture of which baseline data might be useful to measure. We based our parameter selection on a previous review (31).

## Methods

We collected data from June to August 2020 on 55 wards on admitted cases, occupied beds (to control for ward size), involuntary admitted cases, diagnoses, staffing levels (nursing and therapeutic staff) and coercive measures (seclusion, mechanical restraint, and forced medication). The number of assaults was also recorded as early as baseline (see Supplementary Figures S1, S2). Psychiatric wards treating involuntarily admitted patients were eligible to be included into the study. Wards mainly treating people with dementia/delirium were excluded.

To reduce differences in reporting behavior, only physical assaults on people (not property damage or verbally aggressive behavior) were recorded. Primarily, data were provided by medical controlling. If medical controlling could not provide the routine data needed, data were provided by the wards. For this purpose, we used data input tables provided by the study team (see Supplementary Figure S1). All data apart from staffing and occupied beds were provided monthly. Average staffing and occupied beds were recorded at least once.

The proportion of involuntarily admitted cases was approximated by relating each patient involuntarily admitted case present on the ward in each month to admissions and average lengths of stay (see Supplementary material).

In addition, the PreVCo score was assessed once on each ward during an interdisciplinary workshop with ward staff by the PreVCo implementation consultants. The aim of this assessment was to get a more objective impression of the guideline adherence of previous work practices. The assessment of the current degree of implementation achieved with respect to 12 implementation recommendations was conducted by the implementation consultants together with the respective ward teams in a consensus process. Estimates could range from 0 (= not implemented at all) to 9 (= fully implemented) for each recommendation on a Likert scale with anchor examples for each recommendation for the scores 0, 3, 6, and 9 (see Supplementary material). The PreVCo score was then calculated by summing up the points given for each recommendation. Complex interventions that were composed by many interventions (e.g., Weddinger Modell, Safewards, and Six Core Strategies) were multiplied by four. The PreVCo Rating Tool has a possible range from 0 to 135 points.

The wards were randomized to intervention or waiting-list control groups by a 1:1 matched randomization: the participating wards were matched in pairs following the best-fit principle according to the two baseline criteria *frequency of coercive measures per bed and month* and *PreVCo Rating Tool*. The wards were sorted into four groups with a similar number of coercive measures per group. Within their group, they were ranked according to the PreVCo Rating Tool. In this unambiguous ranking, the neighboring wards made up a pair via 1:1 randomization; one ward was randomized to the intervention group, while the other was randomized to the waiting-list control. The matched pair wards did not belong to the same hospital in order to avoid spill-over effects.

We planned to recruit and analyze 52 wards (26 pairs). Since the magnitude of the effect was not known for formal sample size estimation, the case count estimation is based on a realistic estimation of feasibility. The realistic number of wards participating in a nationwide study is assumed to be about 50. Using a paired two-tailed *t*-test, with the primary outcome as the continuous variable, the significance level of 5% and a power of >80%, an effect size of 0.6 can be detected with this case number (nQuery 8.1 Professional, exactly: 24 pairs = 48 wards). Since data are probably not normally distributed, the Wilcoxon signed-rank test for paired data should be used as the evaluation method. This requires about 5%–10% more observations (or rather pairs) to compensate for the loss of power. Accordingly, a case number of 26 pairs (=52 wards) was specified (see study protocol for details, 3). Finally, we recruited all 55 wards that met the inclusion criteria and gave consent to participate. Because of the odd number of participants, one ward was not included in the RCT and 27 pairs were evaluated in this.

To assess for possible differences at baseline between groups, we used Wilcoxon signed-rank tests. The significance level was set to 5%. Because of the exploratory character of this analyses no adjustment for multiple testing was made. For this calculation, we only included the 54 matched wards; for all other calculations, we also included the non-matched ward. To assess correlations between variables, Spearman’s Rho correlation coefficients were calculated.

## Results

The 55 participating wards were staffed with 12.8–25.1 nurses and 0.8–5.3 doctors/psychologists (full-time equivalents) (Table 1). The wards had between 6 and 30 occupied beds per month (median 19) and between 11 and 132 cases were admitted per month (median 41). The percentage of involuntarily admitted cases ranged from 3.4% to 88.9% (median 19.9%). Between 0 and 83 coercive measures per month (median 19), 0–5.8 coercive measures per occupied bed (median 1.0) and 0–2.5 coercive measures per admission (median 0.5) were reported. There were between 0 and 33 assaults in total per month (median 6), 0–1.8 assaults per occupied bed (median 0.3) and 0–1.8 assaults per admission (median 0.1).

**Table 1:** Baseline sample description (*n* = 55).

	**Median**	**Interquartile range (IQR)**	**Minimum; maximum**
*Main diagnosis per month*			
F0	1.67	2.92	0; 11
F1	7.5	12.83	0; 95.67
F2	14	12.25	0.67; 46.33
F3	4.83	5.42	0.33; 27.67
Of whom manic	1	1.92	0; 5.33
F4	3	3	0; 13
F5	0	0	0; 1
F6	2.33	3.58	0; 11.33
F7	0.17	1.33	0; 7
F8	0	0	0; 2
F9	0	0	0; 0.33
Occupied beds	18.7	6.36	5.66; 29.63
Admissions per month	41	25.33	10.67; 131.67
Involuntary admitted cases (%)	19.93	21.16	3.36; 88.9
Coercive measures in total per month	19.33	31.33	0; 82.67
Coercive measures per month and bed	1	1.45	0; 5.77
Coercive measures per admission	0.5	0.61	0; 2.5
Assaults in total per month	6	9	0; 33
Assaults per month and bed	0.3	0.58	0; 1.84
Assaults per admission	0.14	0.27	0; 1.78
Staff nurses (full-time equivalents)	16.14	3.09	11.79; 25.08
Staff doctors/psychologists (full-time equivalents)	3.35	1.25	0.8; 5.3
Seclusion per month	3	11.67	0; 71.33
Cumulative duration (h)	21.6	206.1	0; 998.91
Restraint per month	8	15	0; 67.33
Cumulative duration (h)	51	111.36	0; 2153.98
Forced medication per month	1.67	2.67	0; 16.67

The PreVCo Rating Tool varied between 28 and 106 points. There were considerable differences not only between the wards, but also between the individual items of the rating tool. The median for the individual items ranged between 0 (employment of peers) and 8 (continuous care during a restraint measure), with a possible range of 0–9 points (Table 2).

**Table 2:** PreVCo Rating Tool per item at baseline (range per item 0–9, total range 0–135, *n* = 55, see Supplementary material for description of items).

	Median	IQR	Minimum; maximum
PreVCo Rating Tool	65	22	28; 106
Recording coercion and assaults	6	2	3; 9
Internal standards	7	3	2; 9
Team meetings	3	1	0; 9
Staff training	7	3	0; 9
Continuous supervision during seclusion/restraint	8	4	0; 9
Mandatory debriefing	3	3	1; 8
Peer involvement	0	3	0; 9
Environment	5	3	1; 9
Risk assessment	3	2	0; 9
Advance directives	3	3	0; 8
Pharmacotherapy	7	2	3; 9
Complex interventions (multiplied by four)	2	5	0; 8

**Figure 1:**
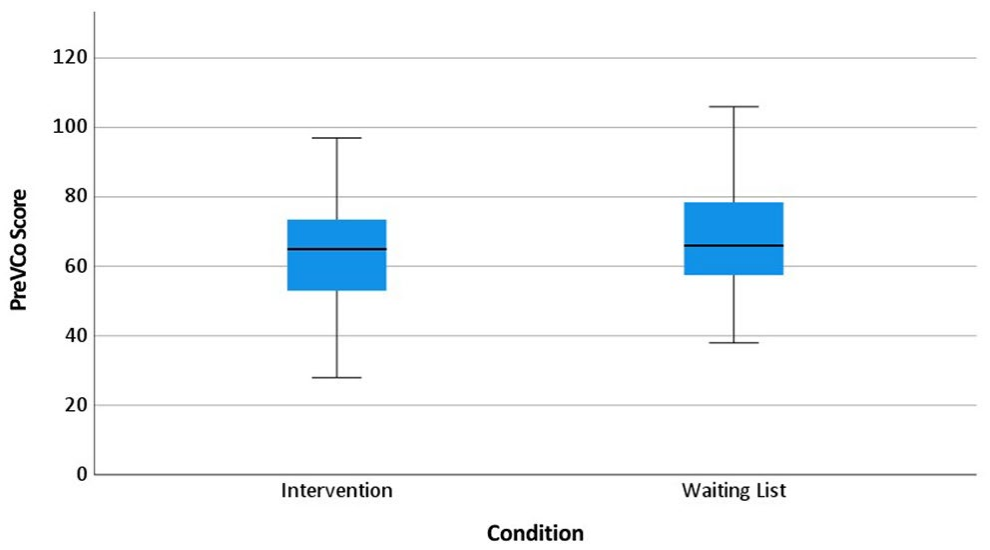
Coercive measures per month and occupied bed at baseline per condition.

**Figure 2:**
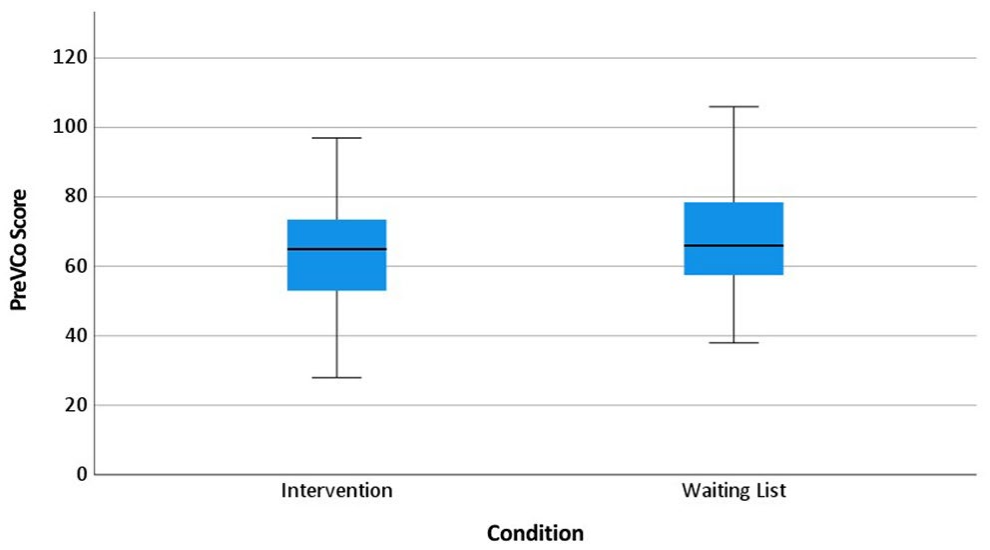
PreVCo Rating Tool score at baseline per condition.

**Figure 3:**
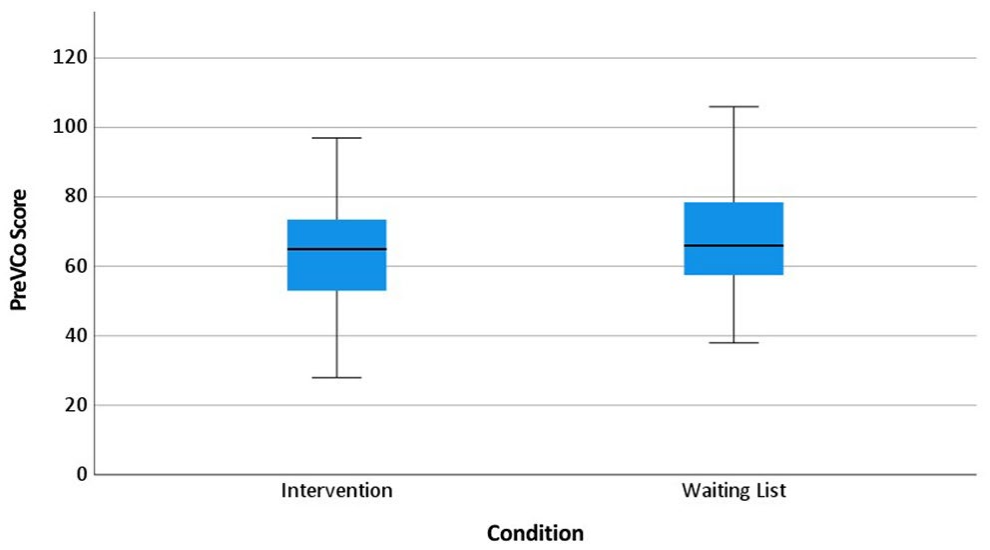
Assaults per month and occupied bed at baseline per condition.

The randomization led to two similar groups which were only significantly different according to assaults (Table 3, Figures 1–3, boxplots) showing more assaults in the waiting list group.

**Table 3:** Baseline sample description per condition (*n* = 54).

*N* = 54	Waiting list			Intervention			Wilcoxon signed rank test
	Median [IQR]	Mean (SD)	Minimum; maximum	Median [IQR]	Mean (SD)	Minimum; maximum	*p*-value
Coercive measures in total per month and occupied bed	0.98 [1.71]	1.36 (1.17)	0.09; 5.12	0.96 [1.34]	1.34 (1.31)	0.00; 5.77	0.442
Cumulated duration of coercive measures per month and occupied bed (h)	6.61 [29.23]	15.81 (19.46)	0.23; 78.39	7.22 [12.97]	12.82 (18.43)	0.00; 90.82	0.61
Admissions per month	37.33 [18.00]	43.88 (25.76)	10.67; 131.67	45.33 [26.33]	48.30 (21.95)	14.33; 105.00	0.319
Occupied beds	18.70 [7.33]	19.64 (4.73)	10.30; 28.50	18.33 [6.12]	18.25 (4.88)	5.66; 27.68	0.414
Staff nurses (full-time equivalents)	16.38 [3.05]	17.13 (2.86)	12.80; 25.08	15.90 [3.12]	16.17 (2.43)	11.79; 22.71	0.361
Staff physicians/psychologists (full-time equivalents)	3.28 [1.40]	3.33 (0.95)	0.80; 5.20	3.54 [1.23]	3.52 (0.91)	1.94; 5.30	0.556
PreVCo Rating Tool	66.00 [23.00]	66.67 (16.84)	38.00; 106.00	65.00 [26.00]	63.85 (16.93)	28.00; 97.00	0.184
Involuntarily admitted cases (%)	0.20 [0.23]	0.27 (0.21)	0.03; 0.89	0.20 [0.10]	0.24 (0.15)	0.05; 0.64	0.737
Assaults per month and occupied bed	0.34 [0.57]	0.55 (0.54)	0.00; 1.84	0.23 [0.57]	0.23 (0.34)	0.00; 1.18	0.049

Ward size, staffing and guideline fidelity at baseline were not statistically significantly correlated with coercive measures per month or occupied bed. Involuntarily admitted cases showed correlation with coercive measures per month and bed (Spearman’s rho = 0.56, *p* < 0.01) as well as the cumulative duration of the coercive measures (rho = 0.41, *p* < 0.01). Assaults showed correlation with coercive measures per month and occupied bed (rho = 0.54, *p* < 0.01), their cumulative duration (rho = 0.54, *p* < 0.01) and with involuntary admitted cases (rho = 0.32, *p* < 0.05). Unsurprisingly, nursing staffing levels were correlated with occupied beds (rho = 0.57, *p* < 0.01) and admissions (rho = 0.33, *p* < 0.05), which is regulated by law in Germany (Table 4).

**Table 4:** Correlation of the key figures at baseline (Spearman’s Rho, *n* = 54).

*N* = 55	Coercive measures in total per month and occupied bed	Cumulated duration of coercive measures per month and occupied bed (h)	Admissions per months	Occupied beds	Staff nurses (full-time equivalents)	Staff physicians/psychologists (full-time equivalents)	PreVCo Rating Tool	Involuntarily admitted patients (%)	Assaults per month and bed
Coercive measures in total per month and occupied bed	–								
Cumulated duration of coercive measures per month and occupied bed (h)	0.40^**^	–							
Admissions per months	0.20	−0.05	–						
Occupied beds	−0.18	−0.09	0.18	–					
Staff nurses (full-time equivalents)	0.01	−0.02	0.33^*^	0.57^**^	–				
Staff physicians/ psychologists (full-time equivalents)	−0.11	−0.08	0.16	0.26	0.17	–			
PreVCo Rating Tool	0.04	0.09	0.00	0.01	0.19	0.20	–		
Involuntarily admitted cases (%)	0.56^**^	0.41^**^	−0.03	0.15	0.24	0.00	0.10	–	
Assaults per month and bed	0.54^**^	0.33^*^	−0.07	−0.16	−0.02	−0.20	0.00	0.32^*^	–

^*^ *p* < 0.05, ^**^ *p* < 0.01.

## Discussion

It is already known from other publications that the number of restraints often differs more between different hospitals and wards than between different countries and care systems (28).

The included wards differed substantially in size (measured by number of beds, staff and admissions), proportion of involuntarily admitted cases, current guideline adherence and number of aggressive incidents. There was also a wide variation in the number of coercive measures applied. Interestingly, there were wards that did not have any coercive measures at all, so ceiling effects must be considered when interpreting the RCT post-intervention. For further RCTs, this should be taken into account and care should be taken already during the recruitment of wards whether they can benefit with regard to the chosen intervention and the chosen endpoint. In addition, the wards differed in other aspects, such as their concept and treatment focus. For example, emergency units, intensive care units and treatment wards were included, but so were wards with a geriatric focus (e.g., wards treating people over 65 years of age with depression or psychosis) or focus on addiction.

The data collection was also affected by the COVID-19 pandemic which increased the differences between wards further (e.g., some wards had to reduce beds) although we shifted the baseline period from spring 2020 to summer 2020. For further RCTs, even when working with aggregated routine data drop-outs must be anticipated.

At least in the cross-sectional assessment, neither the metrics commonly used in the literature (beds per ward, length of stay, allocation of acute patients) were associated with more or less coercion, nor did guideline adherence predict patient-related outcomes (coercion). The RCT as well as the pre-post analyses will show if there are any correlations over time.

The validity of measurement of guideline adherence by the PreVCo Rating Tool might be limited. We tested the feasibility of the rating tool in a pilot study (32) and performed a test for inter-rater reliability (3). However, further psychometric evaluation is pending.

The only measures predicting coercive measures at baseline were the percentage of involuntarily admitted cases and the number of recorded assaults. This is in line with other publications from Germany and elsewhere. In a large-scale study of 136 acute psychiatric wards in England, conflict and coercion were more common when large numbers of patients were placed on wards involuntarily (33), which was in line with expectations. In a registry-based study from the German Land Baden-Württemberg, the final regression model only included the proportion of involuntary cases as a significant predictor, explaining *R*^2^ = 0.27 of the total variance (34). In a study of emergency rooms in Berlin in Germany assaults, involuntary admission, police referral, and younger age were significant predictors for coercive measures (35). PreVCo does not provide data on single patients so we cannot test for referral and age. In a retrospective analysis of involuntary psychiatric admissions in Spain, unscheduled and longer admissions were associated with restraint (36). The PreVCo RCT will show whether there are baseline characteristics which predict better guideline implementation and a reduction in coercion.

The randomization worked well. Except for assaults, there were no significant differences between the intervention and waiting list group at baseline. Therefore, we assumed that good internal validity had been reached. Verifying this, in addition to controlling for observational effects, in particular the Hawthorne effect, was the primary rationale for conducting the baseline survey.

Eventually, the results show that the PreVCo study should provide results with good external validity under real world conditions. The external validity of the results will probably be higher than in the original studies that led to the guideline recommendations. These were often clinical trials in which patients had to give informed consent and in which involuntary patients, who are most likely to be affected by coercion and violence, were not included at all (e.g., 37). In the PreVCo Study, involuntary patients were actually treated in all included wards. Observational effects could be minimized as previously implemented routine data were used to calculate outcome measures and wards did not receive additional funding or staff for study participation.

## Conclusion

The randomization worked well. Our findings that coercion varies widely within a country and is mainly associated with involuntarily admitted and aggressive patients are in line with the international literature. We managed to recruit a large variety of wards and treatment settings. We believe that we were able to include a sample that covers the scope of mental health care practice in Germany well. Therefore, we assume that PreVCo will present with good external validity. The baseline data do not suggest an association of guideline adherence and the amount of coercion.

## Data availability statement

The raw data supporting the conclusions of this article will be made available by the authors, without undue reservation.

## Author contributions

TS and RM designed the study. CC, DS, FB-S collected the data. EF, RM, and SH analyzed the data. SH wrote the first draft. TS and EF contributed to the draft. AB, DS, FB-S, JB, and RM contributed to the final manuscript. TS supervised the project. All authors contributed to the article and approved the submitted version.

## Members of PreVCo study group

Albrecht Schwink, Alexander Menges, Alexandros Michaelides, Andreas Räther, Andrei Vasilescu, Angelika Häusling, Anna Grunze, Anne Möhring, Ann-Kristin Kömmling, Asra Jonuz, Axel Käppeler, Beate Averbeck, Benjamin Grieb, Christian Figge, Christian Frischholz, Christian Kieser, Christoph Neumann, Christoph Richter, Claudia Vallentin, Daniel Schüpbach, Elmar Etzersdorfer, Fabian Franken, Frank Ehrentreich, Friedrich Frieß, Friedrich Jähnel, Hannes Moser, Heinz Grunze, Helmut Vedder, Hubert Lücke, Hubertus Friederich, Ilona Herter, Jakob Löckle, Jana Viehweg, Janina Rix, Jörg Hahn, Julia Seidemann, Kai-James Ludwig, Katharina Kubera, Lorenzo Fenech, Mariana Rudolf, Martin Bürgy, Michael Bauhoff, Patrick Debbelt, Peter Gass, Rene Hurlemann, Sascha Dargel, Susan Ulmer, Susanne McCleskey, Andreas Konrad, Anna Oster, Cordula Sikorski, Eva Béus, Florian Rückert, Jürgen Berg, Katarina Stengler, Marcel Sieberer, Mike Rademacher, Norbert Zumdick, Peter Bräunig, Rudolf Gurnicki, Sabine Bendix, Stefan Lutter, Thomas Scheffel. Thomas Zyzik, Tim Schnitzler, Tobias Rössle, Ulrich von dem Berge, Ulrike Dogue, Uwe Herwig, Vanessa Kielblock.

## Funding

The study is funded by the Innovationsausschuss beim Gemeinsamen Bundesausschuss (Project no. 01VSF19037). The funders had no role in study design or data collection.

## Conflict of interest

The authors declare that the research was conducted in the absence of any commercial or financial relationships that could be construed as a potential conflict of interest.

## Publisher’s note

All claims expressed in this article are solely those of the authors and do not necessarily represent those of their affiliated organizations, or those of the publisher, the editors and the reviewers. Any product that may be evaluated in this article, or claim that may be made by its manufacturer, is not guaranteed or endorsed by the publisher.

## Supplementary material

SUPPLEMENTARY FIGURE S1 Data input tables.

SUPPLEMENTARY FIGURE S2 SPIRIT Flowchart. Reproduced with permission from Steinert T et al. (3), licensed under CC-BY 4.0. (https://creativecommons.org/licenses/by/4.0/).
