# Supplementary figures and images for: Implementation of guidelines on prevention of coercion and violence: baseline data of the randomized controlled PreVCo study
Source: Front Psychiatry. 2023 May 11;14:1130727. doi: 10.3389/fpsyt.2023.1130727 (PMC10213907; doi:10.3389/fpsyt.2023.1130727)

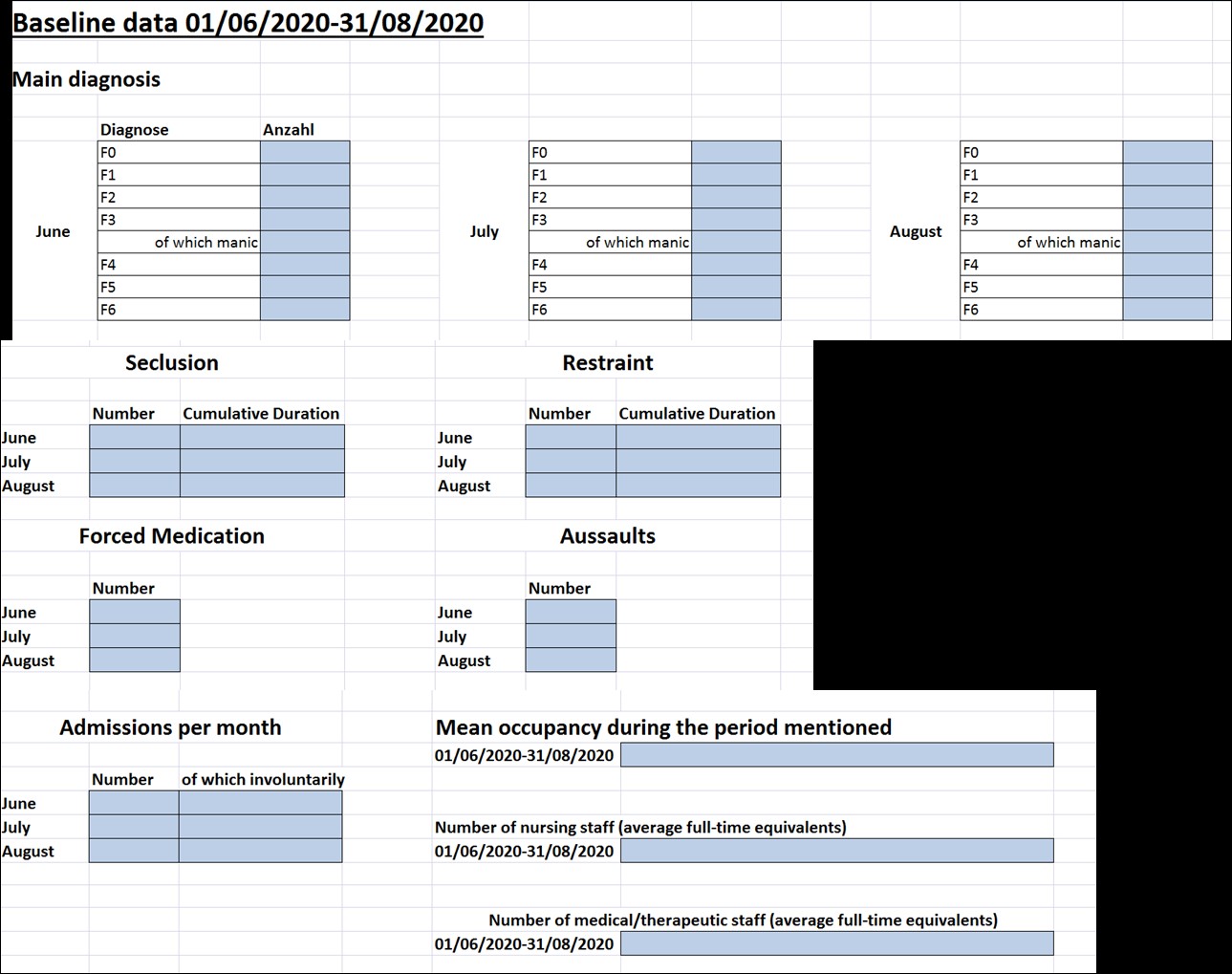

Supplement: SUPPLEMENTARY FIGURE S1 — Data input tables. [file Image_1.JPEG]

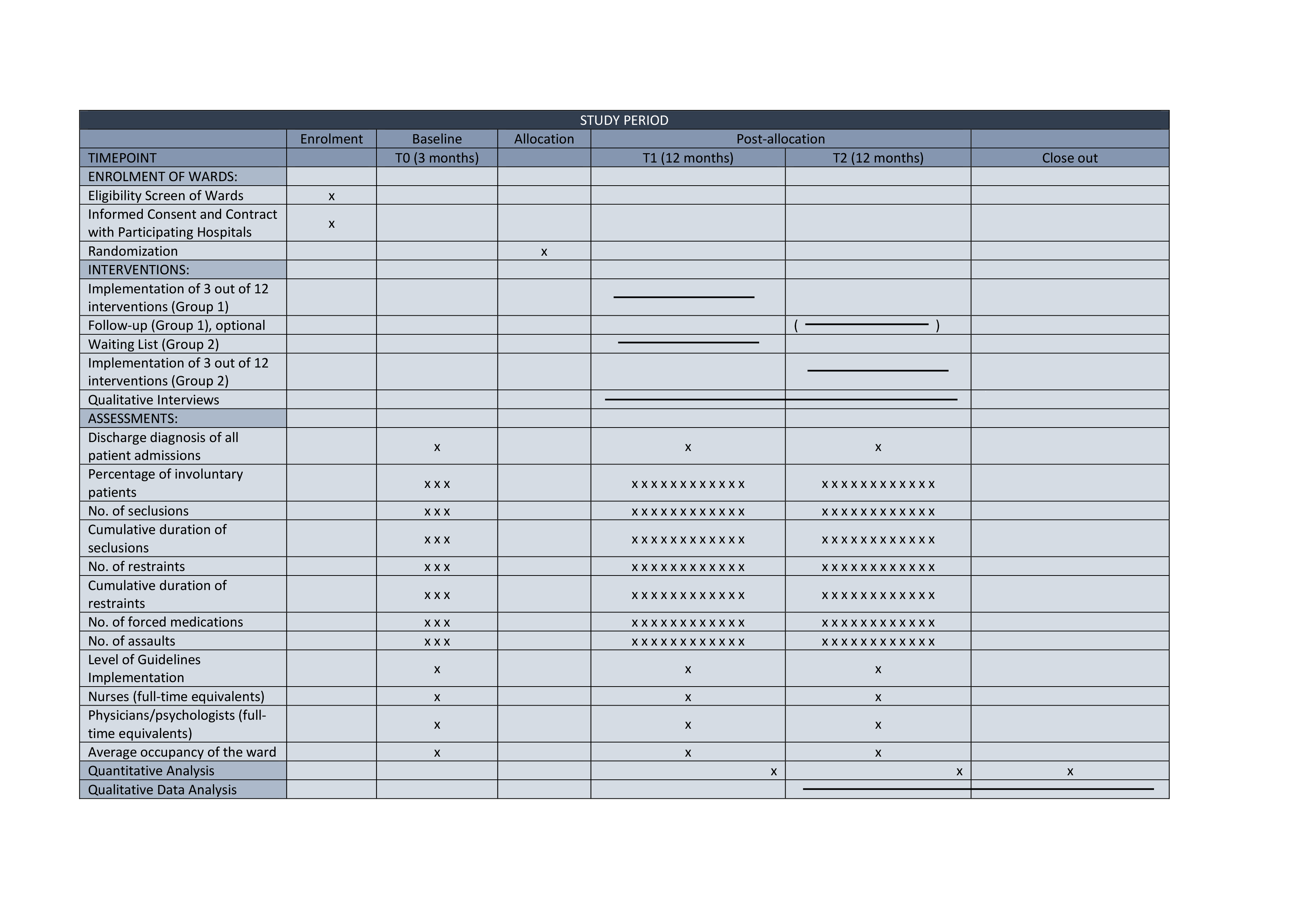

Supplement: SUPPLEMENTARY FIGURE S2 — SPIRIT Flowchart. Reproduced with permission from Steinert T et al. (3), licensed under CC-BY 4.0. (https://creativecommons.org/licenses/by/4.0/). [file Image_2.JPEG]
