# Supplementary material for: Implementation of guidelines on prevention of coercion and violence: baseline data of the randomized controlled PreVCo study
Source: Front Psychiatry. 2023 May 11;14:1130727. doi: 10.3389/fpsyt.2023.1130727 (PMC10213907; doi:10.3389/fpsyt.2023.1130727)
Supplement: Supplementary file 3 [file Data_Sheet_1.PDF]

Supplement:

Proportion of Involuntarily Admitted Cases

The proportion of involuntarily admitted cases was approximated by relating each patient involuntarily present on the ward each month to admissions and average lengths of stay using the following formula:

Mean length of stay (LoS) is calculated by dividing the average number of occupied beds per month (B) by the number of admissions (A) and taking times 30 (all months are assumed to have 30 days for this purpose).

$$LoS = (B/A)*30 \text{ [d]}$$

Then, it was determined in how many months a patient with the corresponding length of stay is counted at least (minC) and at most (maxC). For example, a patient who is only there for one day is certainly only counted once, a patient who is there for two to thirty days can theoretically be counted once or twice and a patient who is there for 31 to 60 days can be counted twice or three times. The average counts are given by:

$$C = minC*(x/30) + maxC*((30-x)/30),$$

With $x = (30+1)-LoS$ resp. $x = (60+1)-LoS$

The quotient of involuntary admissions (corrected by a factor of 1/C) by total admissions is then formed.

PreVCo Rating Tool – 12 Implementation Recommendations Rated on Likert Scales

1. Implement standardized recording of coercive measures and aggressive assaults with opportunity for regular evaluation at the ward level.

(0) No documentation introduced.

(3) Partial documentation on paper, ambiguities regarding operationalization; or one area well regulated, the other not at all.

(6) Both coercion and aggressive assaults are regularly recorded, but some problems remain in operationalization and/or evaluation.

(9) Both coercion and aggressive assaults are regularly and completely recorded in the electronic medical record in a well-operationalized form; a monthly evaluation can be routinely prepared without much effort; operationalization and objectivity of the data are good.

2. Implement internal standards adapting the German clinical practice guidelines.

(0) No internal guidelines or standards available.

(3) Standards/guidelines only rudimentary, e.g., for a limited area (e.g., carrying out restraints); or some standards exist, but not systematized, little known, unclear for whom valid, not generally available or known.

(6) In-house guidelines valid for all professions exist but are poor (in terms of depth or detail, timeliness, availability, familiarity to all practitioners and acceptance in practice).

(9) Internal guidelines valid for all professional groups available that correspond to the status of the clinical practice guidelines; they are readily available, accessible and receives clinical acceptance.

3. Implement a monthly team meeting, led by the department or unit manager, to analyze restraint data and aggressive incidents and discuss background

(0) No such meetings take place; or they take place very rarely and on an ad hoc basis.

(3) Meetings take place occasionally, unsystematically and in changing composition.

(6) The topic is discussed within the multi-professional ward team (at least physicians and nurses) on a regular and scheduled basis, but not monthly or without using their own data.

(9) There are fixed monthly appointments with participation of at least physicians and nurses, where the topic is discussed with reference to their own data. Results and mandatory action orders are derived.

4. Implement a de-escalation/aggression management training plan for all employees with patient contact and ensure that all employees receive appropriate training at least once every two years.

(0) There are no such trainings or trainings are offered on a voluntary basis and occasionally attended by several employees of the ward.

(3) There is a defined training concept that describes both de-escalation and the safe application of coercive measures. Numerous, but not all, ward staff have attended the training.

(6) There is a training concept like (3), this contains explicit contents on prevention, crisis management and post-crisis care. The training concept teaches content that is person and recovery oriented and is trauma sensitive. Also, there is a plan for collaboration among stakeholders in the implementation of restraint measures. Many, but not all, of the unit's staff have participated in the training.

(9) There is a clear and consistent training plan according to criteria (3) and (6) based on a training needs assessment. The trainers have undergone extensive training and refresh their knowledge. The concept provides for a training frequency of at least once within two years for all employees with patient contact and includes that new employees receive the training within the first three months. Compliance rate ≥ 80%.

5. Ensure that continuous supervision is provided in the case of coercive measures that restrict freedom (restraint, seclusion).

(0) Continuous personal care does not take place; or continuous care takes place partly through video surveillance or, in the case of restraints, through observation windows; or continuous care takes place, but for several patients at the same time (this would be surveillance).

(3) Continuous personal care always occurs (exceptions are justified) but, for example, only for restraints (not for seclusions).

(6) Continuous personal supervision by qualified staff always takes place in the case of restraint and seclusion (in the case of restraint at a maximum distance of a few meters, in the case of seclusion via observation windows). Reasons for (rare) exceptions and their documentation are clearly regulated.

(9) As in (6), but intensive care is understood as accompanying the person through the crisis; continuous care serves as far as possible to satisfy needs, promote hope and establish a working alliance.

6. Ensure that mandatory debriefings take place with the affected patients after coercive measures and that they are recorded.

(0) No debriefings take place.

(3) Debriefings are offered in more than half of the cases, but further details of implementation and documentation are not regulated; or there are clear rules for implementation and documentation, but debriefings take place in less than half of the cases.

(6) Debriefings are offered regularly, the content is regulated and the outcome is documented.

(9) Patients who have been subjected to coercive measures are offered regular debriefing sessions, which are conducted in accordance with guidelines (i.e., they include a discussion of different perspectives, allow the patient to recount the situation, offer explanations by staff and record possible complications) and documented and agreements are made to prevent future coercive measures. The attending physician and a primary caregiver participate, as well as other persons of trust or persons involved in the coercive measure at the patient's request.

7. Employ or involve peers to assist in recovery on the ward.

(0) There is no employment or involvement of peers in any way.

(3) There is an option for the involvement of peers, but most patients do not use it or are not aware of e.g., access to counseling; or there is already experience with the involvement of peers in the last 2 years (e.g., internship on the ward), but it is currently not realized.

(6) Peers are available to all patients of the ward on request (i.e., also patients being treated involuntarily), e.g., consultation hours on the ward.

(9) Peers are part of the treatment team (also part-time), peers are involved in the concept development of the ward.

8. Create an action plan for aggression-reducing design of the physical environment on the ward and review it annually.

(0) No such plan exists.

(3) There are plans and initiatives for appropriate and attractive environmental design, but not specifically with regard to the prevention of aggression (e.g., design options regarding pictures, interior furnishings, etc.). The facilities are well-maintained and in good condition.

(6) There were concrete initiatives for the ward in regard to aggression-reducing design of the environment and the facilities, which were also realized, but not in the form of a concrete, written plan. The facilities are well-maintained and in good condition. Rooms reserved for defined purposes (e.g., relaxation room, screen for media) are used.

(9) Annual written plan with the participation of several employees of the therapeutic team and with the involvement of the patients, the contents of which are/were predominantly realized; or new construction within the last five years according to the criteria mentioned.

9. Implement risk assessment with the Brøset Violence Checklist or other tool on all at-risk patients based on clinical assessment and ensure that clinical consequences occur as needed.

(0) No risk assessment introduced.

(3) Risk behavior is usually recognized and is the subject of inter-professional discussions, but no systematic risk assessment takes place; or a form is introduced but is rarely used and does not result in specific interventions.

(6) Form is in place and completed regularly, but an identified elevated risk often does not result in any interventions or only standardized interventions.

(9) Risk assessment form is in place and regularly completed, elevated risk scores regularly result in individualized de-escalating and preventive interventions.

10. Recommend that all patients complete a Patient Advance Directive or offer a Treatment Agreement to prevent further coercive measures after a coercive measure has been carried out.

(0) Patient advance directives or treatment agreements to prevent future coercive measures are not in use.

(3) Appropriate forms are in place but rarely offered; or meetings to prevent future coercive measures are held occasionally, but tend to be at the convenience of the people involved and without written agreements.

(6) Appropriate forms are in place and regularly offered to the patient, even after restraints. However, retrieval and adherence to the agreement is still poor; or fewer than approximately 10 such agreements are completed on the unit annually.

(9) A standardized form for treatment agreements to avoid future coercive measures is available, each patient is offered this after the end of a coercive measure as part of the debriefing and the patient receives a copy of the agreement. It is ensured that all employees are aware of the agreement and that it can be found in the patient's file. A procedure for updating is established (e.g., in case of readmission, after each new coercive measure or if the agreement is older than 5 years. Approximately 10 agreements are concluded each year.

11. Implement measures to ensure guideline-compliant pharmacotherapy, oriented to the clinical practice guidelines regarding aggressive behavior, but also the disorder-specific guidelines.

(0) No measures to ensure guideline-compliant pharmacotherapy are implemented.

(3) Physicians in charge know the guidelines and guideline-compliant work is expected but checked.

(6) The senior consultant/specialist in charge explicitly follows guidelines in prescribing medication and in continuing education. Medication that does not conform to guidelines is rare and justified if necessary.

(9) A review of the conformity of treatment with guidelines takes place regularly in a structured form, e.g., once a month as part of the ward round for each patient. All pharmacological treatments regarding aggressive behavior are carried out in accordance with the guidelines. Medication that does not conform to the guidelines is always explicitly justified.

12. Complex Interventions

(0) Such complex interventions are not known on the ward.

(3) Workers on the ward are familiar with a particular model in outline and there is some kind of commitment or dedication to work according to such a model; concrete implementation steps are rudimentary regarding a few individual interventions.

(6) Team members are familiar with the program and committed to the goals. Responsibilities are named and distributed among several staff members; concrete implementations are formulated in a project plan and are being carried out. The program is at least half implemented. The implementation processes are subject to continuous monitoring and evaluations are available for steps that have been introduced for longer than three months.

(9) The program is implemented. All team members feel responsible for sustaining the interventions. There are clear strategies to ensure sustainability.
